# Morphology, Structure, and Optical Properties of Semiconductor Films with GeSiSn Nanoislands and Strained Layers

**DOI:** 10.1186/s11671-017-2429-6

**Published:** 2018-01-19

**Authors:** Vyacheslav Timofeev, Alexandr Nikiforov, Artur Tuktamyshev, Vladimir Mashanov, Michail Yesin, Aleksey Bloshkin

**Affiliations:** 1 A.V. Rzhanov Institute of Semiconductor Physics SB RAS, 13 Lavrentyev Avenue, Novosibirsk, 630090 Russia; 2 National Research Tomsk Polytechnical University, 36 Lenin Avenue, Tomsk, 634050 Russia

**Keywords:** Epitaxy, Diffraction, Scanning tunnel microscopy, Photoluminescence, GeSiSn layer, Nanoislands, Superstructure, Segregation, Band diagram

## Abstract

The dependences of the two-dimensional to three-dimensional growth (2D-3D) critical transition thickness on the composition for GeSiSn films with a fixed Ge content and Sn content from 0 to 16% at the growth temperature of 150 °С have been obtained. The phase diagrams of the superstructure change during the epitaxial growth of Sn on Si and on Ge(100) have been built. Using the phase diagram data, it becomes possible to identify the Sn cover on the Si surface and to control the Sn segregation on the superstructure observed on the reflection high-energy electron diffraction (RHEED) pattern. The multilayer structures with the GeSiSn pseudomorphic layers and island array of a density up to 1.8 × 10^12^ cm^−2^ have been grown with the considering of the Sn segregation suppression by the decrease of GeSiSn and Si growth temperature. The double-domain (10 × 1) superstructure related to the presence of Sn on the surface was first observed in the multilayer periodic structures during Si growth on the GeSiSn layer. The periodical GeSiSn/Si structures demonstrated the photoluminescence in the range of 0.6–0.85 eV corresponding to the wavelength range of 1.45–2 μm. The calculation of the band diagram for the structure with the pseudomorphic Ge_0.315_Si_0.65_Sn_0.035_ layers allows assuming that photoluminescence peaks correspond to the interband transitions between the *X* valley in Si or the Δ_4_-valley in GeSiSn and the subband of heavy holes in the GeSiSn layer.

## Background

The effective light-emitting devices were not presented since Si is the semiconductor with the indirect bandgap, although the silicon photonics devices, such as waveguides [1], photodetectors [2], and modulators [3] were successfully created.

The Sn addition in the matrix of Ge, Si, or GeSi solid solution is one of the approaches for obtaining the direct bandgap semiconductor which is based on the IV group materials. The inclusion of Sn in the Ge lattice reduces the difference between the minima of *Г* and *L* valleys, and GeSn can become a direct bandgap material. As it was shown, the directness for the GeSn cubic lattice arises at the Sn content of about 9% [4, 5]. This value can be lower than 6% in the presence of the tensile strain, whereas for films with a compressive strain, the transition can be expected for the Sn content more than 11% [6]. The bandgap reduction due to the inclusion of Sn allows increasing the operating wavelength from the near to middle infrared range, which is desirable in the field of optical interconnections, in new-generation fiber optic communication systems, sensors, signal processing, energy conversion, and optical storage devices [7]. Therefore, for the recent years, the interest to the class of Ge-Si-Sn materials has significantly increased.

Big efforts are pooled to obtaining the epitaxial GeSn films of instrumental quality [8, 9]. One of the serious problems is the Sn inclusion (precipitates) formation during GeSn layer growth [10, 11]. In addition to precipitation, Sn segregation is observed in the process of GeSn, GeSiSn layer growth, and GeSn film oxidation [11, 12]. Non-equilibrium growth techniques, such as the molecular beam epitaxy (MBE) and chemical vapor deposition (CVD), serve reliable methods of the precipitation and segregation suppression. To reduce the effect of Sn precipitation and segregation, the growth temperature decrease [13] or the deformation inclusion, or the addition of the third element, for example, Si, which reduces the local stress around the Sn atoms, can be performed.

The data on morphology and surface structure or on the formation mechanisms of single-crystal GeSiSn films are, practically, not found in the literature, despite of a great promise of the materials based on group IV compounds. These data are necessary for the creation of nanoheterostructures with strained and relaxed GeSiSn layers. In this paper, the data related to the creation of multilayer structures, superlattices not only with pseudomorphic GeSiSn layers but also with GeSiSn nanoislands, are first demonstrated, and they do not contain dislocations and allow varying the bandgap width of the material in a wide range and cover the infrared wavelength range previously unavailable for GeSi.

The purpose of this paper is to study the elastically stressed pseudomorphic GeSiSn film growth, three-dimensional nanoisland formation and to obtain the multilayer periodical structures containing pseudomorphic GeSiSn layers and nanoislands.

In our previous papers [14–16], the kinetic diagrams of morphological GeSiSn film state were constructed for various lattice parameter mismatches between GeSiSn and Si in the Sn content range from 0 to 8% using the reflection high-energy electron diffraction (RHEED). This article includes new critical two-dimensional to three-dimensional growth (2D-3D) transition thickness dependences on the GeSiSn film composition with a fixed Ge content in the Sn content range from 0 to 16%. The phase diagrams of the superstructure change during the growth of Sn on Si and Ge(100) were obtained. In spite of the fact that in [17–19], Sn surface reconstructions on Si were studied at the different tin covers, our data demonstrated new superstructures. In addition, the phase diagram of the superstructure change during the Sn growth on Ge(100) was not earlier presented anywhere. The optical properties of multilayer periodic structures including pseudomorphic GeSiSn layers were first studied by the photoluminescence method for samples with different Sn contents. The band diagram calculation of GeSiSn/Si heterostructures was carried out using the model solid theory approach [20].

## Experimental

All samples with pseudomorphic GeSiSn layers and with GeSiSn nanoislands were grown at ultrahigh vacuum conditions 10^−7^–10^−8^ Pa on molecular beam epitaxy (MBE) equipment “Katun C.” The epitaxial growth chamber has the electron beam evaporator for Si and the Knudsen effusion cells for Ge and Sn. The GeSiSn layer growth rate was varied from 0.015 to 0.05 nm/s. The epitaxial growth was carried out on Si(100) substrates in the temperature range of 150–450 °С with a Sn content from 0 to 20%. Not only single GeSiSn layers but also multilayer periodic structures, containing GeSiSn/Si heterojunctions, were obtained. At first, the GeSiSn layer was deposited, which then was covered by a 10-nm Si layer at growth temperature 400–500 °С. The main technique of controlling the changes in morphology and surface structure and investigating the growth mechanisms was reflection of high-energy electron diffraction (RHEED). The RHEED pattern was recorded on a video camera during the growth. Then, a profile, along with one of the crystallographical directions, was chosen, and the intensity changes of this profile in space-time coordinates were created. The RHEED space-time intensity distribution analysis allowed us to study the mechanisms of two-dimensional growth, superstructure changes, and three-dimensional island formation. The moment of the transition from the two-dimensional to three-dimensional growth (2D-3D transition) was determined from the time dependence of RHEED pattern intensity along with one of the rods in the direction of which the volume reflex appears. The dependences of critical 2D-3D transition thickness on the GeSiSn content with a fixed Ge content were created based on the method of 2D-3D transition determination. Based on these dependences, the GeSiSn pseudomorphic layer thickness was set in multilayer structures, and layers with a GeSiSn nanoisland array were created. The morphology and surface structure were analyzed by scanning tunneling microscopy (STM) on ultrahigh vacuum equipment Omicron-Riber. The sample optical properties were studied by photoluminescence (PL) spectroscopy using an ACTON 2300i monochromator and a cooled OMA-V detector based on the InGaAs photodiode array with a sensitivity band of 1.1 to 2.2 μm. To excite photoluminescence, the Nd:YAG laser radiation (532 nm) was used.

## Results and Discussion

**Fig. 1:**
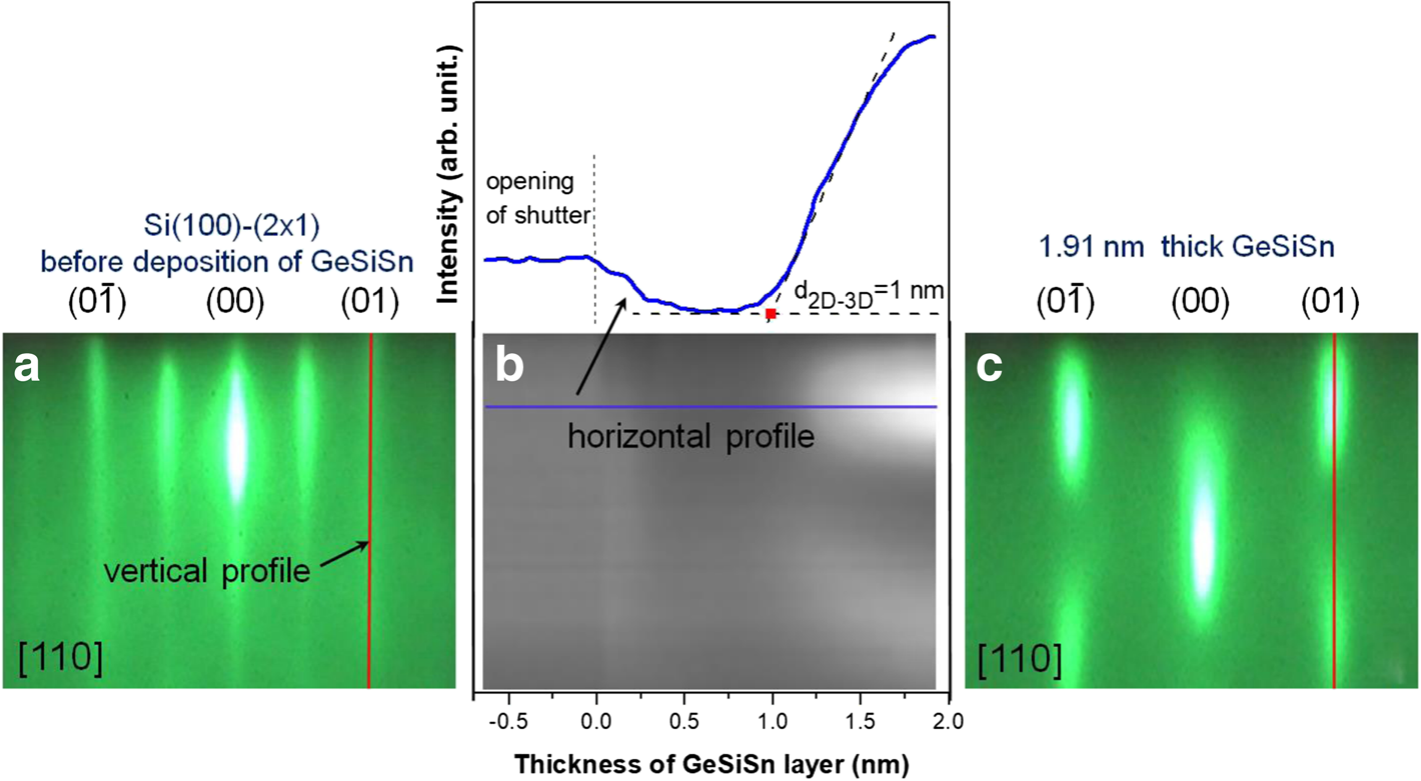
2D-3D transition moment determination during the GeSiSn film growth: **a** RHEED pattern from the Si(100)-(2 × 1) surface before the Ge_0.6_Si_0.28_Sn_0.12_ growth is shown, **b** the space-time intensity distribution of the vertical profile in the gray scale and the intensity dependence of the horizontal profile on the deposited Ge_0.6_Si_0.28_Sn_0.12_ film thickness. The profiles are indicated by the arrows in (**a**) and (**b**), and **c** the final RHEED pattern after the 1.91-nm-thick Ge_0.6_Si_0.28_Sn_0.12_ deposition

The thin GeSiSn film growth at the temperature of 150 °С in the Sn content range from 0 to 16% was studied. The strain accumulation occurs during the GeSiSn layer deposition caused by the mismatch between the GeSiSn and Si lattice parameter. The transition from 2D-3D transition is observed at a certain thickness. The procedure for determining of the 2D-3D transition on the example of the Ge_0.6_Si_0.28_Sn_0.12_ growth is presented in Fig. 1. There are the initial RHEED patterns from the Si surface before the Ge_0.6_Si_0.28_Sn_0.12_ film deposition (Fig. 1a), the final RHEED pattern (Fig. 1c) formed by the Ge_0.6_Si_0.28_Sn_0.12_ island array and the wetting layer, as well as the space-time intensity distribution of the vertical profile indicated by the arrow in Fig. 1a and the dependence of the horizontal profile intensity (horizontal profile indicated on the space-time intensity distribution in Fig. 1b) on the Ge_0.6_Si_0.28_Sn_0.12_ film thickness (Fig. 1b). The 2D-3D transition moment was determined by plotting the tangent to the intensity plot on the thickness (Fig. 1b) in the region of a sharp intensity increase. Such approach is generally accepted [21].

**Fig. 2:**
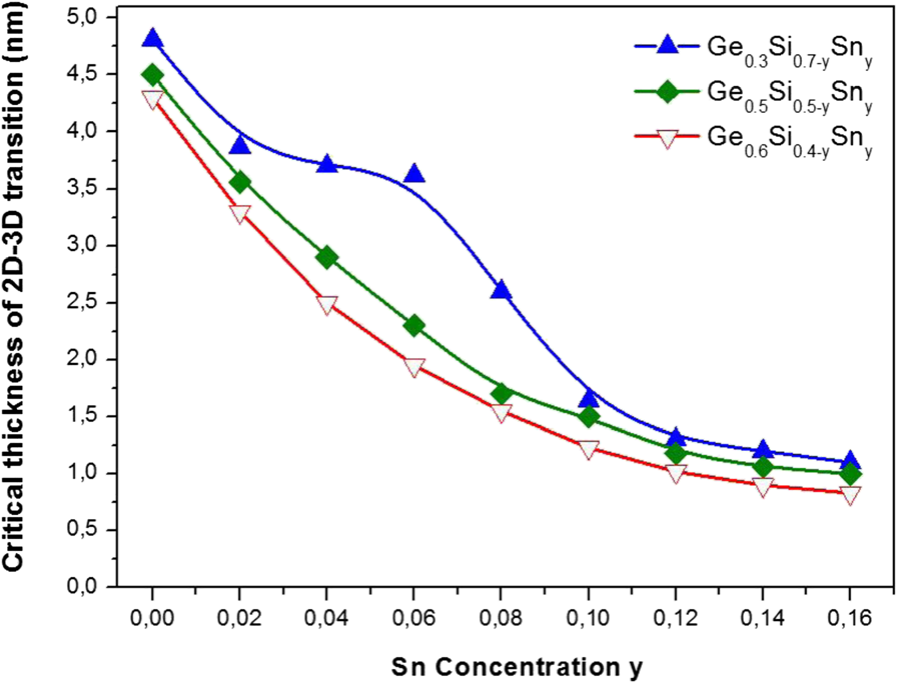
The critical 2D-3D transition thickness dependences on the GeSiSn film composition at several fixed values of Ge content, Sn content from 0 to 16%, and at the growth temperature of 150 °С

The dependences of the critical 2D-3D transition thickness on the composition for GeSiSn films with a fixed Ge content and a Sn content from 0 to 16% are built (Fig. 2) using the 2D-3D transition technique described above. Earlier, the kinetic diagrams for the morphological state of GeSiSn films in the temperature range of 150–450 °С, at a different lattice mismatch between GeSiSn and Si, were published [14]. On the base of kinetic diagram analysis, the optimum temperature of 150 °С was determined, at which the critical 2D-3D transition thickness reaches its maximum value and the Sn segregation is suppressed. The GeSiSn film thickness value below the curve corresponding to the critical 2D-3D transition thickness on the temperature and composition determines the region of the existence of pseudomorphic films. The decrease of the critical 2D-3D transition thickness, with the increase at the Sn content from 0 to 16% is observed on the curves (Fig. 2). Such behavior is explained by the strain effect. The increase of the Sn content from 0 to 16%, for example, the Ge_0.6_Si_0.28_Sn_0.12_ growth, results in the rise of the lattice parameter mismatch between Ge_0.6_Si_0.28_Sn_0.12_ and Si from 2.5 to 5.6%, respectively, and reducing the time and, consequently, the transition thickness to three-dimensional nanoislands. The nature of the feature which appears on the curve with the 30% Ge content and is observed at the Sn content from 3 to 10% to the end is completely not clear. Knowing the magnitude of the critical 2D-3D transition thickness, it is possible to obtain the pseudomorphic GeSiSn films and use them in the multilayer periodic structures with the GeSiSn/Si heterojunction. In our experiments, the accuracy of determining the critical 2D-3D transition thickness is 0.06 nm and it is determined mainly by the inaccuracy of maintaining the Si flow rate due to the source operation instability.

**Fig. 3:**
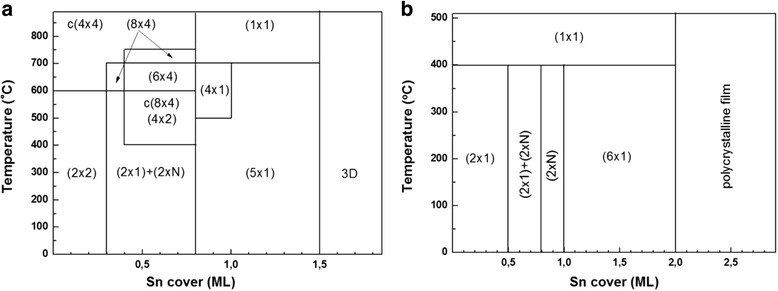
Phase diagrams of the change of the superstructure during growth: **a** Sn on Si(100) and **b** Sn on Ge(100)

The choice of the pseudomorphic layer thickness on the base of the kinetic diagrams obtained earlier [16] and the dependences of the critical 2D-3D transition thickness on the composition (Fig. 2) allow growing not only single GeSiSn layers but also using these layers in multilayer periodic structures. In the periodic GeSiSn/Si structure, where the GeSiSn layer is covered by the Si layer, the problem arises due to the Sn segregation onto the Si surface. The Sn segregation leads to the surface reconstruction and the appearance of the whole series of superstructures depending on the Sn surface concentration. The investigation of the surface reconstruction during the Sn growth on Si(100) and Ge(100) was necessary in order to understand which Sn coating corresponds to the superstructure observed in the RHEED picture. The phase diagrams of the change of the superstructure were built in the temperature range of 100–750 °С. A similar diagram for the Sn growth on Si(100) was first presented in [17]. Ueda et al. deposited Sn at room temperature followed by annealing. They observed the superstructure only after annealing the film. In our experiments, Sn was deposited at the temperature of 100 °С and it was obtained on the reconstructed Si(100–(2 × 1) surface. The temperature increase up to 750 °С resulted in the formation of the superstructure series on the surface (Fig. 3). The position effect of adsorbed Sn atoms on the Si(100) surface on the Sn-Si binding energy was studied by the photoelectron spectroscopy in [18]. The Sn-Si binding energy decrease was observed with the Sn cover increase. Thereby, all the reconstructions, which occur during the Sn growth on Si(100), can be explained by a decrease in the surface system energy. The transition to the three-dimensional growth is associated with the accumulation of strains due to the lattice parameter mismatch between Sn and Si of 19%. The polycrystalline film appearance was observed in [17] at the 3.2 ML (monolayer) Sn thick film. The polycrystalline film formation is caused by a low deposition temperature of the Sn film. The same reasoning is related to the Sn growth on the Ge(100) surface. At present, contributions on the Sn superstructures on Ge(100) have not been reported in the literature.

**Fig. 4:**
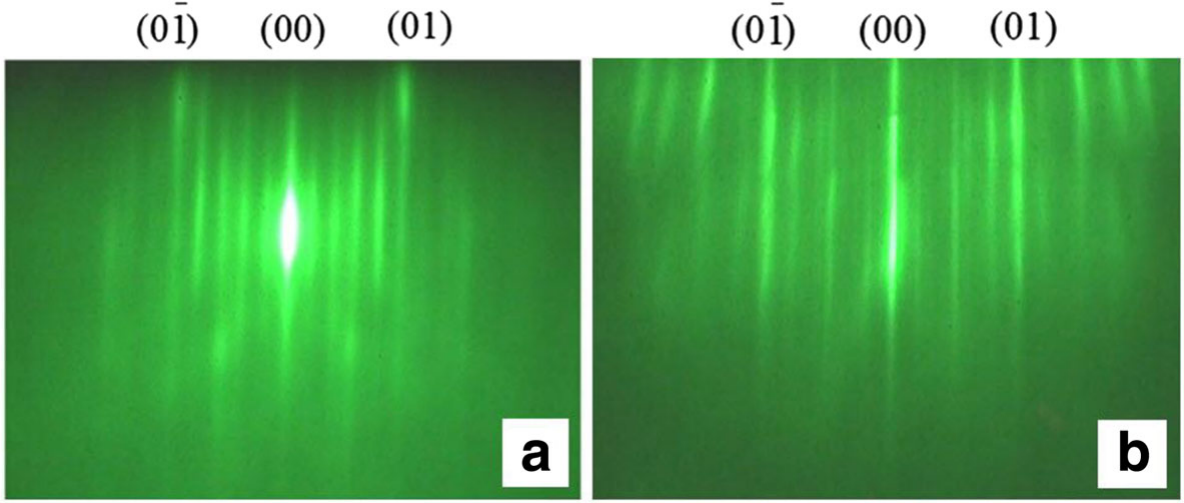
Superstructures observed at the Si growth on the GeSiSn layer in the multilayer periodic structure in the azimuthal [110] direction: **a** (5 × 1) and **b** (10 × 1)

The superstructures indicated in the diagrams appear both with the temperature increase and decrease. These phase diagrams help to determine the Sn concentration on the surface at the Si growth over the GeSiSn layer. The superstructures appearing on the Si surface during the periodic structure growth can be observed at the temperatures which differ from the temperatures presented in the phase diagrams. The Si layer is deposited on the GeSiSn surface at temperatures 400–500 °С; however, the superstructures which are characteristic for the whole temperature range presented in Fig. 3 may appear. The creation of the structure with GeSiSn/Si heterojunctions requires preventing the formation of the two-domain (5 × 1) superstructure, which is associated with the Sn segregation and the obstruction in the multilayer periodic structure formation with the pseudomorphic layers (Fig. 4a). The simplest way to suppress Sn segregation at the Si growth over the GeSiSn layer is the Si growth in two stages. The first stage involves the 1–2-nm-thick Si layer deposition at room temperature. The further Si growth continues at the temperature of 400–500 °С. This temperature is determined by the Sn content in the GeSiSn layer. The main superstructure series that occur during the Si growth on the GeSiSn layer in multilayer periodic structures consist of (2 × 1) + (2 × N), c(8 × 4), (4 × 1), (6 × 1), and (5 × 1). In addition, the two-domain (10 × 1) superstructure is observed on the Si surface (Fig. 4b). This superstructure did not appear in the experiments at the Sn growth on Si and Ge, but it can be said that it corresponds to the minimum Sn coating since it disappears during a short annealing at a temperature of 400–500 °С and the (2 × 1) superstructure appears, which is a characteristic for the Si surface.

**Fig. 5:**
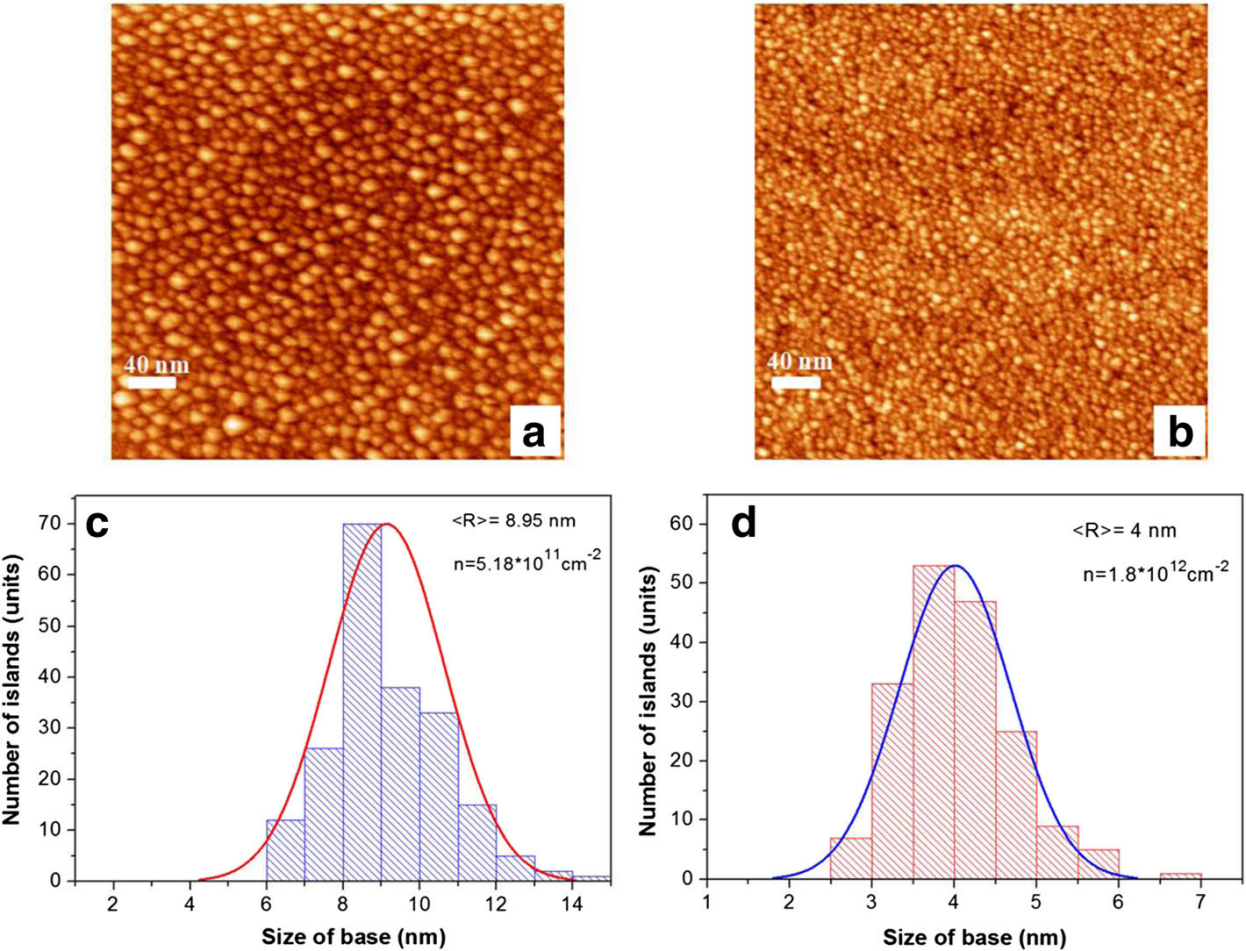
STM images of the Ge_0.75_Si_0.2_Sn_0.05_ surface with the scan size of 400 nm × 400 nm: **a** the Ge_0.75_Si_0.2_Sn_0.05_ surface in the first period, **b** the Ge_0.75_Si_0.2_Sn_0.05_ surface in the fifth period; the distribution histograms for the number of islands on the size of the base for the Ge_0.75_Si_0.2_Sn_0.05_ film: **c** in the first period (the Ge_0.75_Si_0.2_Sn_0.05_ film thickness equals 1.78 nm) and **d** in the fifth period (the Ge_0.75_Si_0.2_Sn_0.05_ film thickness equals 1.89 nm)

Periodic structures, containing pseudomorphic layers or a GeSiSn nanoisland array, were obtained. Using the kinetic diagrams of GeSiSn film morphological state, a GeSiSn island array in the multilayer periodic structure was investigated. The STM pictures of Ge_0.75_Si_0.2_Sn_0.05_ surface with the nanoisland array in the first (Fig. 5a) and in the fifth period (Fig. 5b) with a scan size of 400 nm × 400 nm are presented. The island array was obtained at the growth temperature of 250 °С. The histograms of island number distribution in size are also demonstrated in Fig. 5. The islands with density 5.18 × 10^11^ cm^−2^ and average size 8.95 nm are presented in Fig. 5c. The deposited Ge_0.75_Si_0.2_Sn_0.05_ film thickness was 1.78 nm. The average island size in the fifth period is about 4 nm, and the island density reaches 1.8 × 10^12^ sm^−2^ at an effective thickness of Ge_0.75_Si_0.2_Sn_0.05_ film 1.89 nm, and it follows from the histogram in Fig. 5d. An increase in the density by a factor of 3.5 and a decrease in the island size by a factor of 2 may be related to the increasing Sn fraction on the Si surface with an increase in the period number. This statement is confirmed by the change in superstructures, observed by the RHEED pattern during the Si film growth over the GeSiSn layer, from (2 × 1) and (2 × N) to the c(8 × 4) surface structure. The formation of c(8 × 4) superstructure takes place during the process of Sn growth on Si, starting from the covering thickness of 0.4 monolayer (ML) at growth temperature 400 °С. With the decreasing growth temperature to 100 °С, we can increase the island density, but the surface quality is worse. A growth temperature increase to more than 250 °С enhances the Sn segregation. So, the optimal growth temperature range of GeSiSn layers with the island array is 150–250 °С, where specular reflection oscillations are observed at the GeSiSn wetting layer growth, corresponding to the 2D growth mechanism.

**Fig. 6:**
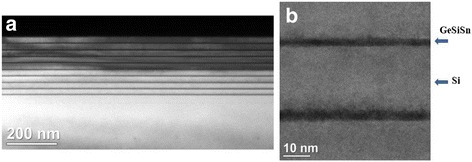
**a** TEM image from the multilayer structure including the Ge_0.5_Si_0.45_Sn_0.05_ heterotransition with the 25-nm period. **b** High-resolution TEM image from the same structure

The crystalline perfection of multilayer structure was studied by transmission electron microscopy (TEM). The TEM images for a multilayer structure with the Ge_0.5_Si_0.45_Sn_0.05_/Si heterojunction and a 25-nm period are shown in Fig. 6. From the TEM data, it can be concluded that our samples do not contain threading dislocations and are crystalline perfect with sharp interfaces. The pseudomorphic GeSiSn film state in a multilayer periodic structure, the crystal lattice, and strains are discussed in [14] on the base of the data analysis obtained with the help of TEM. The GeSiSn film composition was determined setting Si, Ge, and Sn flows. The growth rates of Si, Ge, and Sn were measured with a quartz thickness meter. The GeSiSn film composition was identified by X-ray diffractometry. The analysis showed the coincidence of both the set and measured compositions.

**Fig. 7:**
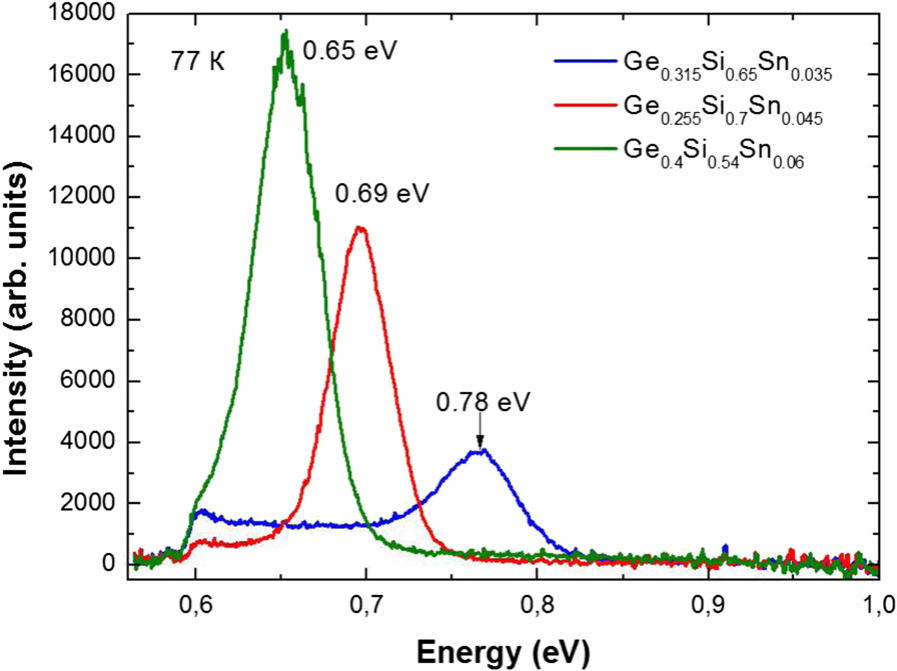
The photoluminescence spectra from multilayer periodic structures with the 3.5, 4.5, and 6% Sn content in the pseudomorphic GeSiSn layers

The optical properties of multilayer periodic structures with GeSiSn layers were investigated by photoluminescence for structures with different Sn contents. The structures demonstrated their photoluminescence in the range of 0.6–0.8 eV, which corresponds to the wavelength range of 1.45–2 μm (Fig. 7). The photoluminescence spectra with the intensity maxima at 0.78, 0.69, and 0.65 eV were obtained. They correspond to the 1.59, 1.8, and 1.9 μm wavelengths, and they are observed at 3.5, 4.5, and 6% Sn, respectively. The Sn content increase in the GeSiSn layer leads to both the energy decrease of optical transitions and the photoluminescence intensity increase. The intensity increase may be caused by the depth increase of the quantum well for a higher Sn content in the GeSiSn solid solution layer. To advance in the wavelength range at more than 2 μm, an increase of the Sn content in GeSiSn layers is required. To determine the optical transitions observed in the luminescence spectra, it was necessary to calculate the band diagram of the GeSiSn/Si heterostructure.

The GeSiSn/Si heterostructure band diagram was calculated using the approach of model solid theory [20]. Within this model, both semiconductors forming the heterojunction need to put to the single “energy” scale to determine the band position in the heterojunction. The valence bandgap value for Ge/Si heterostructures in the heterojunction is well known [22] and equals 0.54 eV; moreover, the Ge valence band lies higher on the energy than the Si valence band. The bandgap between Ge and Sn is known from the model presented in [23] and is 0.69 eV [24]. Thus, the bandgap value on the heterointerface for the Si/Si_*1-x-y*_Ge_*x*_Sn_*y*_ heterostructure can be written as:

$$ {E}_{v, av}=0.54x+1.23y $$

Since the semiconductor valence band is formed by the subbands of heavy and light holes, and also by the subband splitted off by the spin-orbit interaction; then, the position of the valence band (*E*_*v,av*_) averaged in the three subbands is used to determine the bandgap and construction of the heterostructure band diagram. To determine the heavy and light hole subband position, as well as the subband splitted off by the spin-orbit interaction, the following expressions are used:

$$ {\displaystyle \begin{array}{l}{E}_{\mathrm{HH}\left(\mathrm{LH}\right)}={E}_{v, av}+{\Delta}_0/3\\ {}{E}_{\mathrm{SO}}={E}_{v, av}-2/3{\Delta}_0\end{array}}, $$

where the HH, LH, and SO indices indicate the heavy and light hole subbands, as well as the spin-splitted subband. The Δ_0_ value denotes the spin-orbit splitting energy in the semiconductor. After determining the valence band subband position in the heterojunction, the positions of the corresponding conduction band valleys are determined using the expression:

$$ {E}^n={E}_{\mathrm{Ge}}^n\left(1-x-y\right)+{E}_{\mathrm{Si}}^nx+{E}_{\mathrm{Sn}}^ny-{b}_{\mathrm{Si}\mathrm{Ge}}^nx\left(1-x-y\right)-{b}_{\mathrm{Sn}\mathrm{Ge}}^ny\left(1-x-y\right)-{b}_{\mathrm{Si}\mathrm{Sn}}^n xy, $$

where indexes *n* = *Γ*, *L*, and *X* indicate the corresponding valleys ${b}_{\mathrm{SiGe}}^n$, ${b}_{\mathrm{SnGe}}^n$, and ${b}_{\mathrm{SiSn}}^n$––“bowing” parameters, taking into account the deviation from the linear law for the bandgap width, ${E}_{\mathrm{Ge}}^n$,${E}_{\mathrm{Si}}^n$, and ${E}_{\mathrm{Sn}}^n$––Ge, Si, and Sn band gaps in the corresponding valley. Almost all parameters are taken from [24]. The bowing parameters for valleys *L* and *Г* are taken from [25].

After determining the position of all bands of interest in the heterojunction, we took into account their displacement under the influence of deformations. The influence of deformations on the band gap was taken into account by means of deformation potential constants [26]. Since the two-dimensional layers were pseudomorphic, in our case, the standard approach was used to determine deformations which is described, for example, in [26]: strains in a quantum well plane can be determined from *ε*_*xx(yy)*_ = *ε*_ǀǀ_ = (*a*_GeSiSn_ − *a*_Si_)/*a*_Si_, where *a*––the lattice constant of the corresponding material. In the direction, which is perpendicular to the plane of the quantum well, strain value *ε*_*zz*_ = −2(*С*_12_/*С*_11_)*ε*_*xx*_ can be determined through the *C*_12_ and *C*_11_ elastic modules of the crystalline compound. The solid solution lattice constant was determined from the quadratic relation:

$$ {a}_{\mathrm{Ge}\mathrm{SiSn}}={a}_{\mathrm{Ge}}\left(1-x-y\right)+{a}_{\mathrm{Si}}x+{a}_{\mathrm{Sn}}y+{b}_{\mathrm{Si}\mathrm{Ge}}^{\hbox{'}}x\left(1-x\right)+{b}_{\mathrm{Sn}\mathrm{Ge}}^{\hbox{'}}y\left(1-y\right), $$

where *a*_Ge_, *a*_Si_, *a*_Sn_––Ge, Si, and Sn lattice parameters [24], ${b}_{\mathrm{SiGe}}^{\hbox{'}}$= − 0.026 Å, ${b}_{\mathrm{SnGe}}^{\hbox{'}}$ = 0.166 Å––«bowing» parameters, taking into account the deviation from Vegard law.

**Fig. 8:**
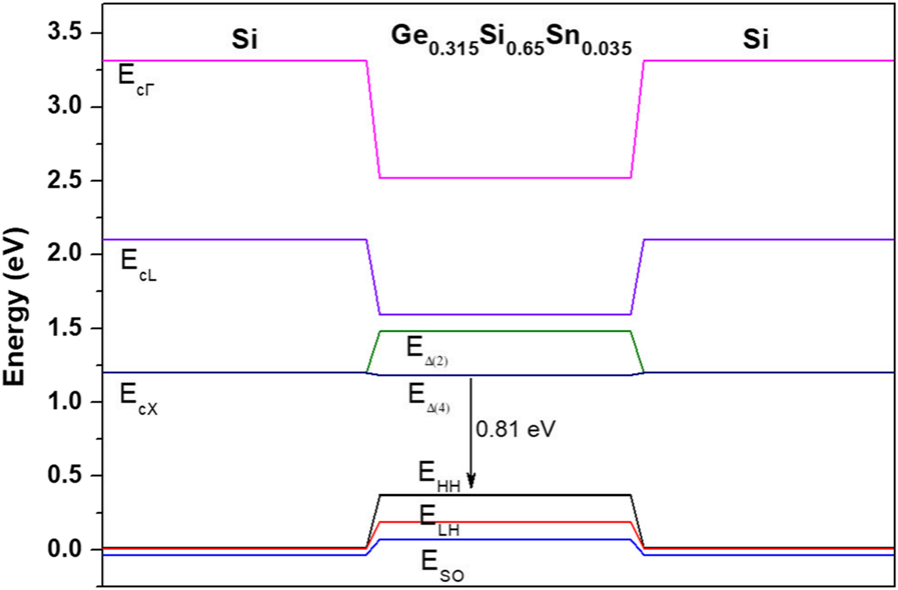
The Si/Ge_0.315_Si_0.65_Sn_0.035_/Si heterocomposition band diagram

Based on the band diagram calculations, the PL peaks correspond to the interband transitions between the X-valley of Si or the Δ_4_-valley of GeSiSn and the heavy-hole band in the GeSiSn layer (Fig. 8).

## Conclusions

The critical 2D-3D transition thickness dependencies on the composition of GeSiSn layers with a fixed Ge content and Sn content, varying from 0 to 16% at the growth temperature of 150 °С, were determined. The superstructure phase diagrams for the epitaxial growth of Sn on Si(100) and Ge(100) were made. The multilayer periodic structures with pseudomorphic layers and with a GeSiSn island array of a density up to 1.8 × 10^12^ sm^−2^ were obtained. For the first time, in the multilayer periodic structure during the Si growth on the GeSiSn layer, a (10 × 1) two-domain superstructure, which transforms into a (2 × 1) superstructure at a slight annealing, was observed. The GeSiSn/Si periodic structures demonstrated their photoluminescence in the range of 0.6–0.8 eV, which corresponds to the wavelength range of 1.45–2 μm. Based on the band diagram calculations for the structure with pseudomorphic Ge_0.315_Si_0.65_Sn_0.035_ layers, the PL peaks correspond to the interband transitions between the X-valley of Si or the Δ_4_-valley of GeSiSn and the heavy-hole band in the GeSiSn layer. Further progress in the long-wave region requires an investigation of the optical properties of the structures with a large Sn content.

## Abbreviations

CVD: Chemical vapor deposition
MBE: Molecular beam epitaxy
ML: Monolayer
PL: Photoluminescence
RHEED: Reflection high energy electron diffraction
STM: Scanning tunneling microscopy
TEM: Transmission electron microscopy

## References

[1] Xia F, Sekaric L, Vlasov Y (2007). Ultracompact optical buffers on a silicon chip. Nat Photonics.

[2] Assefa S, Xia F, Vlasov Y (2010). Reinventing germanium avalanche photodetector for nanophotonic on-chip optical interconnects. Nature.

[3] Xu Q, Schmidt B, Pradhan S, Lipson M (2005). Micrometre-scale silicon electro-optic modulator. Nature.

[4] Du W, Ghetmiri SA, Conley BR, Mosleh A, Nazzal A, Soref RA, Sun G, Tolle J, Margetis J, Naseem HA, SQ Y (2014). Competition of optical transitions between direct and indirect bandgaps in Ge_1-x_Sn_x_. Appl Phys Lett.

[5] Senaratne CL, Gallagher JD, Aoki T, Kouvetakis J, Menéndez J (2014). Advances in light emission from group-IV alloys via lattice engineering and n-type doping based on custom-designed chemistries. Chem Mater.

[6] Wirths S, Buca D, Mantl S (2016). Si–Ge–Sn alloys: from growth to applications. Prog Cryst Growth Charact Mater.

[7] Soref RA (2008). Toward silicon-based long wave integrated optoelectronics (LIO). Proc SPIE.

[8] Wirths S, Tiedemann AT, Ikonic Z, Harrison P, Hollander B, Stoica T, Mussler G, Myronov M, Hartmann JM, Grutzmacher D, Buca D, Mantl S (2013). Band engineering and growth of tensile strained Ge/(Si)GeSn heterostructures for tunnel field effect transistors. Appl Phys Lett.

[9] Von den Driesch N, Stange D, Wirths S, Mussler G, Hollander B, Ikonic Z, Hartmann JM, Stoica T, Mantl S, Grutzmacher D, Buca D (2015). Direct bandgap group IV epitaxy on Si for laser applications. Chem Mater.

[10] Takeuchi S, Sakai A, Nakatsuka O, Ogawa M, Zaima S (2008). Tensile strained Ge layers on strain-relaxed Ge_1-x_Sn_x_/virtual Ge substrates. Thin Solid Films.

[11] Kato K, Asano T, Taoka N, Sakashita M, Takeuchi W, Nakatsuka O, Zaima S (2014). Robustness of Sn precipitation during thermal oxidation of Ge(1-x)Sn(x) on Ge(001). Japan J Appl Phys.

[12] Taoka N, Asano T, Yamaha T, Terashima T, Nakatsuka O, Costina I, Zaumseil P, Capellini G, Zaima S, Schroeder T (2015). Non-uniform depth distributions of Sn concentration induced by Sn migration and desorption during GeSnSi layer formation. Appl Phys Lett.

[13] Tsukamoto T, Hirose N, Kasamatsu A, Mimura T, Matsui T, Suda Y (2015). Investigation of Sn surface segregation during GeSn epitaxial growth by Auger electron spectroscopy and energy dispersive x-ray spectroscopy. Appl Phys Lett.

[14] Timofeev VA, Nikiforov AI, Tuktamyshev AR, Mashanov VI, Gutakovskii AK, Baidakova NA (2016). Strained multilayer structures with pseudomorphic GeSiSn layers. Semiconductors.

[15] Nikiforov AI, Mashanov VI, Timofeev VA, Pchelyakov OP, Cheng HH (2014). Reflection high energy electron diffraction studies on SixSnyGe1-x-y on Si(100) molecular beam epitaxial growth. Thin Solid Films.

[16] Tuktamyshev AR, Mashanov VI, Timofeev VA, Nikiforov AI, Teys SA (2015). Initial growth stages of Si-Ge-Sn ternary alloys grown on Si(100) by low-temperature molecular-beam epitaxy. Semiconductors.

[17] Ueda K, Kinoshita K (1984). Study of superstructures on Sn/Si(100) by means of RHEED-LEED-AES. Surf Sci.

[18] Rich DH, Miller T, Samsavar A, Lin HF, Chiang TC (1988). Adsorption and growth of Sn on Si(100) from synchrotron photoemission studies. Phys Rev B.

[19] Wakahara A, Vong KK, Hasegawa T, Fujihara A, Sasaki A (1995). Surfactant effects of Sn on SiGe/Si heteroepitaxy by molecular beam epitaxy. J Cryst Growth.

[20] Van de Walle CG (1989). Band lineups and deformation potentials in the model-solid theory. Phys Rev B.

[21] Simon L, Louis P, Pirri C, Aubel D, Bischoff JL, Kubler L, Bolmont D (2003). Substrate manipulation by insertion of a thin and strained 2D layer: effect on Ge/Si growth. J Cryst Growth.

[22] El Kurdi M, Sauvage S, Fishman G, Boucaud P (2006). Band-edge alignment of SiGe/Si quantum wells and SiGe/Si self-assembled islands. Phys Rev B.

[23] Jaros M (1988). Simple analytic model for heterojunction band offsets. Phys Rev B.

[24] Moontragoon P, Soref R, Ikonic Z (2012). The direct and indirect bandgaps of unstrained SixGe1−x−ySny and their photonic device applications. J Appl Phys.

[25] Fischer IA, Wendav T, Augel L, Jitpakdeebodin S, Oliveira F, Benedetti A, Stefanov S, Chiussi S, Capellini G, Busch K, Schulze J (2015). Growth and characterization of SiGeSn quantum well photodiodes. Opt Express.

[26] Attiaoui A, Moutanabbir O (2014). Indirect-to-direct band gap transition in relaxed and strained Ge1−x−ySixSny ternary alloys. J Appl Phys.

